# Actin Cytoskeleton and Regulation of TGFβ Signaling: Exploring Their Links

**DOI:** 10.3390/biom11020336

**Published:** 2021-02-23

**Authors:** Roberta Melchionna, Paola Trono, Annalisa Tocci, Paola Nisticò

**Affiliations:** 1 Tumor Immunology and Immunotherapy Unit, IRCCS Regina Elena National Cancer Institute, via Chianesi 53, 00144 Rome, Italy; roberta.melchionna@ifo.gov.it (R.M.); paola.trono@ifo.gov.it (P.T.); annalisa.tocci@ifo.gov.it (A.T.); 2 Institute of Biochemistry and Cell Biology, National Research Council, via Ramarini 32, 00015 Monterotondo Scalo, Rome, Italy

**Keywords:** actin cytoskeleton, actin-binding proteins, TGFβ, extracellular matrix, tumor microenvironment

## Abstract

Human tissues, to maintain their architecture and function, respond to injuries by activating intricate biochemical and physical mechanisms that regulates intercellular communication crucial in maintaining tissue homeostasis. Coordination of the communication occurs through the activity of different actin cytoskeletal regulators, physically connected to extracellular matrix through integrins, generating a platform of biochemical and biomechanical signaling that is deregulated in cancer. Among the major pathways, a controller of cellular functions is the cytokine transforming growth factor β (TGFβ), which remains a complex and central signaling network still to be interpreted and explained in cancer progression. Here, we discuss the link between actin dynamics and TGFβ signaling with the aim of exploring their aberrant interaction in cancer.

## 1. Introduction

Actin dynamics critically affect different aspects of human health and disease, ranging from embryonic development to wound repair, inflammation, and cancer [1]. Each of these complex processes requires that a coordinated formation of multiple actin-based structures occurs. Actin dynamics refer to dynamic rearrangements of actin-based structures whose spatial and temporal control generates the physical force that enables cell motile functions. Almost all the cells, from prokaryotic to multicellular organisms, have the ability to sense a wide range of physical and chemical signals from the surrounding environment and elaborate specific responses by adjusting their cytoskeletal organization, and hence their shape and motility [2].

A fine regulation of actin filament dynamics guarantees plasticity and dynamicity of actin cytoskeleton that allows the cell to elaborate the adequate cytoskeleton-based responses to stimuli. Although for a long time considered merely regulators of cell mechanical properties, actin dynamics have emerged as platforms for signaling pathways [3]. Notably, actin dynamics have been linked to gene transcription, pointing out mechanisms that communicate the cytoplasmic actin status to the nuclear genome [4].

The transforming growth factor β (TGFβ) family encompasses cytokines with widespread and diverse biological effects. Essentially, all cells perceive TGFβ family signals and elaborate responses that affect cell proliferation, differentiation, communication, adhesion, movement, metabolism, and death. The wide-ranging nature of biological signals elicited by TGFβ underlies the complexity of this pathway, which mediates fate decisions during development, tissue homeostasis and regeneration, tumorigenesis, fibrosis, and the immune response [5,6,7,8,9,10,11].

The effects of the cytokines of TGFβ family are mediated by a combinatorial set of ligands and receptors and can differ dramatically depending on the cellular context [12]. The TGFβ signaling pathway is critically influenced by mechanical cues including extracellular matrix (ECM) stiffness, cell–cell adhesion, cell–matrix adhesion, cell density, cell tension and cell polarity [13] all processes guaranteed by the plasticity and dynamicity of actin cytoskeleton.

While the role of TGFβ signaling in the organization of actin cytoskeleton architecture has been extensively investigated, the ways in which actin cytoskeleton dynamics modulate TGFβ signaling have just started to emerge.

Herein, we provide a brief overview of the actin dynamics and TGFβ signaling pathway and present evidence to disclose their interaction, starting from ECM, through the plasma membrane up to the nucleusall steps where actin and TGFβ signaling crosstalk and affect cancer.

## 2. Actin Cytoskeleton: Architecture and Signaling

### 2.1. Actin Cytoskeleton Architecture and Dynamics

Actin, one of the most abundant and highly conserved proteins in eukaryotic cells, is a 42 kDa adenosine-5′-triphosphate (ATP)-binding protein that cycles from monomeric globular actin (G-actin) to polymeric filamentous actin (F-actin) in a complex process governed by many different actin-binding proteins (ABPs) [14].

Actin polymerization gives rise to filaments with different types of organization: branched and cross-linked networks, parallel bundles, and anti-parallel contractile structures located in specific subcellular compartments [15]. Branched and crosslinked networks of lamellipodia at the front of the cell represent the major engine of cell movement, along with aligned bundles of filopodia. A thin layer of actin forms the cell cortex at the plasma membrane, contributing to the maintenance of cell shape. Interspersed in the rest of the cell, the actin scaffold is made up of a three-dimensional network of cross-linked filaments and contractile bundles, including stress fibers that connect the cell cytoskeleton to the extracellular matrix via focal adhesion sites. Incorporated within the actin network are filaments of the molecular motor protein myosin that produce contraction in the cell body and tension at focal adhesion [15].

This organization makes the cytoskeleton highly responsive to stimuli and regulates dynamic cell behavior [1]. The activity balance of actin assembly factors has to be achieved to ensure the main functions of the cytoskeletal system: to generate force, to create structural scaffolds, and to act as tracks for motor proteins [16]. At a molecular level, actin filament turnover proceeds through finely tuned steps: monomer sequestration or delivery, filament nucleation, elongation, capping, severing, or depolymerization [14]. The rate and direction of polymerization, as well as the shape of the newly generated filament, are determined by local intracellular concentrations of ATP-bound G-actin and by the activity of ABPs [14].

### 2.2. Actin Cytoskeleton Regulatory Proteins

ABPs act as key players of actin dynamics, participating in diverse processes ranging from maintaining a large pool of actin monomers available for polymerization, to initiating polymerization, nucleating assembly of new filaments, promoting elongation, capping the ends of polymers to terminate elongation, severing, and crosslinking of filaments [14]. G-actin-binding proteins maintain high concentrations of free G-actin by preventing spontaneous nucleation of new filaments while barbed end capping proteins prevent their elongation.

Profilin binds weakly to ATP-actin at the barbed ends, catalyzes nucleotide exchange by rapidly dissociating adenosine-5′-diphosphate (ADP) from newly depolymerized actin monomers, allowing further barbed end elongation [17]. To maintain the actin monomer pool, profilin cooperates with the capping protein (CP) [18]. Furthermore, profilin promotes elongation of barbed ends by delivering actin to polyproline sequences of proteins such as formins and Ena/VASP. At the same time, profilin prevents spontaneous actin nucleation and elongation at the actin pointed ends [14].

To initiate actin filament polymerization in a controlled manner, cells use an Arp2/3 complex to produce actin filament branches, formins to initiate unbranched filaments, and proteins with tandem WH-2 domains to form other filaments [14].

Severing proteins such as cofilin and members of the gelsolin family promote actin filament turnover by disassembling actin filaments, and producing free barbed ends. A large family of cross-linking proteins operate to physically connect actin filaments and stabilize higher-order structures, such as bundles of filaments in microvilli, filopodia, and cytoplasmic cables, as well as networks of actin filaments [19,20].

Among the actin cytoskeleton regulatory proteins, we focus on Ena/VASP, critical players in metastatic cancer progression. Ena/VASP proteins promote actin elongation via antagonizing actin filament capping [21]. The Ena/VASP family encompasses three mammalian family members: VASP (vasodilator-stimulated phosphoprotein), MENA (mammalian ENA homolog), and EVL (Ena-VASP-like), sharing a conserved domain structure. Each family member consists of an N-terminal EVH1 domain, a central proline-rich region, and a C-terminal EVH2 domain [22]. The EVH2 domain, which contains G-actin and F-actin-binding sites, is responsible for promoting actin polymerization [23,24,25]. In contrast, the EVH1 domain mediates intracellular targeting of Ena/VASP proteins by interacting with the “FPPPP” repeats of diverse proteins, including zyxin [26,27], and determines the recruitment of Ena/VASP proteins to specific sites within the cell [22]. The central proline-rich region harbors binding sites for SH3 and WW domain-containing proteins and the actin monomer-binding protein profilin [28]. Ena/VASP proteins are localized to the leading edge of lamellipodia and the tips of filopodia, but also to stress fibers and focal adhesions [28,29]. MENA is the only family member that contains a unique long insertion close to EVH1 domain of five-amino acid long stretch of highly charged basic and acidic amino acids (LERER) [28]. Notably, MENA binds to the C-terminal end of the cytoplasmic tail of the integrin α5 via LERER domain, affecting α5β1 signaling [30]. Differently from all other members of Ena/VASP, there are multiple splice variants of the MENA gene, enabled homolog (*ENAH)*, which are involved in several mechanisms critical for cancer progression. *ENAH* contains a small coding exon 11a that is included only in epithelial cells and excluded in mesenchymal cell lines and downregulated during epithelial-mesenchymal transition (EMT) [31,32,33]. The splicing of this exon is mainly regulated by the Epithelial Splicing Regulatory Proteins 1 and 2 (ESRP1 and ESRP2) [33]. In invasive tumor cells, the hMENA^11a^ downregulation occurs, and the human MENA (hMENA) splice variant lacking the internal exon 6 (hMENAΔv6) is upregulated [34]. ENAH splice variants containing alternative exons ++ and +++ (Mena INV), linked to cancer cell invasiveness, have also been described [35,36]. Our group has identified the two alternatively expressed isoforms, hMENA^11a^ and hMENAΔv6, that exert opposite roles in breast, lung, and pancreatic cancer cell invasiveness [34,37,38]. Their role during cancer progression and their crosstalk with TGFβ signaling will be discussed in Section 4 and Section 5.

### 2.3. Actin Dynamics and Signaling: From ECM to the Nucleus

Cell–matrix contacts are dynamic, and cells sense external force and make adjustments, by modulating the cytoskeleton filaments and their linkages to transmembrane proteins [39]. At sites of cell-ECM adhesion, cells sense physical features of the microenvironment through integrins and other adhesive proteins and adjust their own tensional state through actomyosin contractility and organization of the F-actin cytoskeleton to counterbalance extracellular forces [39,40]. Thus, the mechanotransduction process relies on force generation by actin-myosin networks and force transmission through adhesive complexes. Actomyosin contractility is the result of contractile force predominantly generated by the action of myosin II motors on actin filaments. The RhoA and RhoC family members promote actomyosin contractile force generation through ROCK1- and ROCK2-mediated phosphorylation of different downstream target proteins, including LIM kinases 1 and 2 (LIMK1 and LIMK2), the myosin regulatory light chain (MLC) [41]. Force transmission at adhesive complexes relies on the activation of integrins which are clustered into macromolecular structures associated with plasma membrane at the cytoplasmic side, namely focal adhesions, that provide a direct physical link between intracellular actin cytoskeleton and ECM. Integrin activation results from a conformational reorganization of the α-β integrin dimer that critically increases its affinity to the matrix ligand. Proteins such as talins and kindlins bind to β integrin cytoplasmic domains, connecting them to the actin cytoskeleton [2]. A reciprocal regulation between integrins and F-actin exists: integrins promote bundling of actin filaments to generate tension within a cell. Conversely, actin regulatory proteins, actin polymerization, and spatial organization affect integrin function [39].

By playing a key part in the matrix-sensing machinery, actin cytoskeleton, for a long time considered the skeleton of the cell, now emerges as a platform for signal transduction [4], able to transmit tensile stresses generated at the interface between the cell and ECM to the nucleus, thus affecting nuclear properties and gene expression programs [42].

LINC (linker of nucleoskeleton and cytoskeleton) complex is one illustrative player of the transduction of mechanical signals to the nucleus through cytoskeletal physical links [43]. LINC complex, located at the nuclear envelope (NE), enables dynamic rearrangements of the cytoskeletal actin to be communicated to the nucleus. The nuclear membrane proteins Nesprins are components of the LINC complex and sustain the physical connection between the cytoskeleton and nuclear lamina, allowing cells to maintain tension between the plasma membrane and nucleus, essential for both organelle and cytoskeletal organization [44]. Nesprins are characterized by a C-terminal transmembrane domain, the KASH-domain, which anchors them in the nuclear membrane and a N-terminal domain by which they connect the nucleus to the actin cytoskeleton (Nesprin-1 and -2). Connections within the nucleus are mediated by nuclear lamina (lamins A, B1, B2, and C) and associated proteins like emerin.

To the actin cytoskeleton-mediated nuclear transduction also contribute transcription factors such as Yes-associated protein (YAP) and Myocardin-Related Transcription Factors (MRTFs), whose shuttling between the cytoplasm and the nucleus through the nuclear pore complex (NPC) is regulated by actin dynamics.

YAP and TAZ (transcriptional co-activator with PDZ-binding motif) are critical players in the sensing and transduction of mechanical cues to gene expression [45]. They elicit biological effects in response to ECM elasticity by acting as transcriptional cofactors that shuttle from the cytoplasm to the nucleus [45]. YAP and TAZ play key roles not only in physiological settings, but also in tumorigenesis, cancer stem cell induction, cancer-associated fibroblast (CAF) activation, and chemoresistance [46,47]. YAP/TAZ localize to the nucleus and are transcriptionally active in cells plated on large and stiff substrates, which display high ROCK-and non-muscle-myosin-II-driven cytoskeletal tension. Conversely, YAP/TAZ are excluded from the nucleus when cells are plated on softer or smaller substrates, which impose cell rounding and reduced adhesive area [48]. Noteworthy, YAP/TAZ activity is regulated by the conformation and tension of the F-actin cytoskeleton. Rather than F-actin total levels, YAP/TAZ is regulated by the F-actin subcellular organization, fine structure, tension, and resistance offered by molecular structures within the cytoskeleton and by the whole nucleus [48].

The MRTF-SRF circuit is activated by the Rho GTPase family, which regulates essentially every aspect of actin cytoskeleton function [49]. Rho GTPases actively participate in processes by which actin dynamics are transmitted up to the nucleus by regulating effector proteins that modulate the equilibrium of G-actin and F-actin in the cytoplasm. Actin polymerization, downstream to Rho GTPases thus affects the MRTF-SRF (serum response factor) circuit. In the presence of high levels of cytoplasmic G-actin, MRTFs, able to bind G-actin, are retained in the cytoplasm. Changes in actin dynamics may imply the incorporation of G-actin into the F-actin filaments, causing a reduction of G-actin concentration, thus leaving MRTFs free to enter the nucleus and to interact with the transcription factor SRF, which drives the expression of its target genes [50].

It is important to mention that actin also exists within the nucleus, where it is involved in transcription, chromatin remodeling and intra-nuclear movements [51,52]. Although implicated in a variety of different nuclear processes, the underlying mechanisms still remain unclear.

## 3. TGFβ Biology and Signaling in Cancer

Herein, we briefly summarize basics of TGFβ biology and signaling that are covered more extensively in excellent reviews [53,54,55,56,57].

### 3.1. Brief Overview of TGFβ Isoforms and Their Role in Normal and Pathological Context

Transforming growth factor β factors (TGFβ1, TGFβ2, and TGFβ3) are multi-tasking cytokines implicated in the regulation of a broad range of cellular functions and different biological processes i.e., embryogenesis, immune regulation, fibrosis-associated diseases, and tumor progression [12,58].

Encoded by different genes and uniquely expressed in mammals [59], TGFβ isoforms exert peculiar and not-redundant functions in vivo, as suggested by the diversity of phenotypes of knockout mice for each isoform [60,61,62,63]. TGFβ ligands, alone or in the context of other environmental cues, balance the self-renewal and differentiation process of stem cells and define cell fate during embryonic lineage specification and differentiation [8]. A body of evidence has indicated TGFβ factors as crucial in controlling the immune system [64], during development and maturation of immune cells, in maintaining immune tolerance and homeostasis and controlling innate, as well as in the adaptive immune system [65,66,67]. Moreover, context-dependent pro- or anti-inflammatory effects have been clearly demonstrated [67].

Following tissue damage, TGFβ1 becomes a master regulator of the repair process, providing a rapid restoration of tissue integrity, by reprogramming and suppressing excessive tissue inflammation [58,64,68,69,70].

The deregulation of TGFβ activity may shift the physiological repair to pathological condition such as fibrosis, an aberrant repair response which strongly affects organ functionality. TGFβ implication in the pathogenesis of fibrosis-associated diseases has been extensively sustained by experimental and in vivo models as well as clinical evidence [71,72,73,74,75]. On the other hand, therapeutic implementation of anti-TGFβ approaches in fibrosis-associated diseases is still hampered by the pleiotropic multifunctional role of TGFβ, leaving opened many questions to design effective therapies [58].

In cancer, TGFβ exerts both tumor-suppressive and tumor-promoting functions, referred to as “TGFβ Paradox”, which represents the most critical and cryptic issue of the physio-pathological TGFβ role [76,77]. To suppress tumor, TGFβ induces apoptosis in pre-malignant cells and inhibits cancer cell proliferation, whereas in the late stage of tumorigenesis, it sustains tumor progression. The switch from tumor-suppressive to tumor-promoting function of TGFβ is intimately linked to the triggering of EMT program, closely related to actin cytoskeleton modifications, as further detailed.

In the tumor microenvironment, stromal cells are an abundant source of TGFβ [78], with cancer-associated fibroblasts (CAFs) the most significant producers [79,80]. Activated TGFβ signaling leads to CAF activation, CAF-mediated cancer progression [81] and may differently influence CAF subsets [82].

Noteworthy, CAF-derived TGFβ may contribute to immunosurveillance and escape and may participate in therapy resistance, including immunotherapy with immune checkpoint blockade [83,84,85], also by excluding CD8+ T lymphocytes from the tumor site [86,87]. A recent paper suggests that TGFβ neutralization targets only selected CAF subtypes and, in turn, promotes CAF immunomodulatory properties and the formation of an immune-permissive tumor microenvironment (TME) by regulating ECM density [82].

### 3.2. Basics of TGFβ Signaling

TGFβ ligands signal to the nucleus, mainly through serine/threonine kinase receptors tetrameric complexes composed of two type II receptors (TβRII) and two type I receptors (TβRI). The receptor activation initiates signaling via both canonical SMAD2/3 and non-canonical pathways.

In the canonical TGFβ-SMAD pathway, upon TGFβ binding, TβRII trans-phosphorylates TβRI and activates its kinase activity resulting in phosphorylation and mobilization of SMAD proteins which include SMAD1, 2, 3, 5 and 8 [88]. SMAD2/3 phosphorylation is required for their association with SMAD4 in mammalian cells [89]. This heterotrimeric complex translocates into the nucleus where it activates or represses the transcription of several genes [88,90]. The interaction of SMAD protein complex with other transcription factors and/or with additional co-activators and co-repressors as well as with chromatin-modifying enzymes ensures the context-dependent cellular response to TGFβ signaling [57].

In the non-canonical pathways, TGFβ can activate the mitogen-activated protein kinases (MAPKs), the extracellular signal-regulated kinase (ERK), phosphatidylinositide 3-kinase (PI3K)/AKT, TNF receptor-associated factor 4/6 (TRAF 4/6) and Rho-like GTPases (Rho) [56,91,92,93,94,95]. Notably, TGFβ has been reported as able to crosstalk with several other signaling pathways (i.e., Wnt, Notch, Hippo signaling, and with tyrosine kinase receptors) to elicit a context-dependent signaling [12,96].

## 4. TGFβ-Induced Actin Reorganization in Physio-and Pathological Contexts

The actin cytoskeleton reorganization is one of the earliest cellular response to TGFβ signaling, which may generate rapid and long-term modifications of actin dynamics, thus modulating morphology, adhesion, growth, motility, and invasiveness of different cell types. In both non-transformed and transformed epithelial cells, TGFβ can rapidly induce membrane ruffling and actin polymerization at the cell edges [97], whereas a prolonged stimulus results in stable actin filament bundles (stress fibers) [98]. In the short-term response, TGFβ induces rapid activation of GTPases Rho family, including Rac, Cell Division Cycle 42 (CDC42), and RhoA [98], followed by ROCK⁄LIMK⁄cofilin pathway activation and actin cytoskeleton polymerization [99]. On the contrary, the long-term actin cytoskeleton response involves the SMAD pathway, and leads to transcriptional regulation of RhoB and α-SMA in fibroblasts [100].

TGFβ induces myofibroblast differentiation by increasing Rho-dependent cytoskeletal stress fiber formation and Rho/actin/MRTF-A/SRF signaling pathway [101].

TGFβ signaling controls cellular plasticity and is essential in EMT promotion, a physiological process crucial in tissue and organ formation during development, but also acts as a facilitator of tumor progression [102]. Remodeling of the cytoskeleton is a hallmark of EMT, and notably, the actin regulatory genes are the most highly upregulated during TGFβ-induced EMT [103,104,105,106,107]. Several actin cytoskeleton-associated proteins, including hMENA and hMENAΔv6, have been reported to be upregulated during EMT [38,103,104,105,108,109]. Functionally, as a consequence of the TGFβ-induced cytoskeletal remodeling, actin stress fibers are formed in cancer cells, affecting cell shape and function and favoring cancer cell invasion and tissue rigidity [107].

## 5. Actin-Related Regulatory Functions that Control TGFβ Signaling

**Figure 1:**
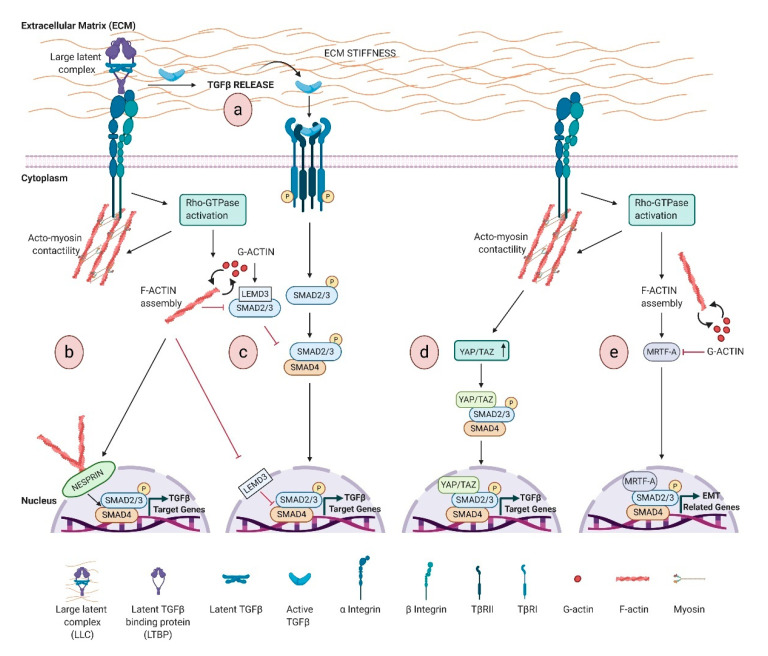
Schematic representation of transforming growth factor β (TGFβ) signaling regulation by actin remodeling induced by extracellular matrix (ECM) stiffness. Activation of Rho GTPase by mechanical cues promotes F-actin assembly and actin cytoskeleton contractility, which activates TGFβ signaling by: (**a**) Inducing TGFβ ligand release and activation. The ECM-integrin-actin cytoskeleton linkages allow integrin to shift toward an active configuration that favors TGFβ release from LTBP/latent TGFβ complex. Active TGFβ initiates signaling via TGFβ receptors, which ultimately drives the phosphorylation-dependent formation of SMAD2/3-4 complex. This complex translocates to the nucleus and facilitates the transcription of TGFβ-dependent genes; (**b**) Accumulating at the front of the nucleus of the lamin-binding protein Nesprin-2, which induces SMAD nuclear localization; (**c**) Inhibiting the formation of both cytosolic and nuclear LEMD3-SMAD2/3 complexes, resulting in the relief of LEMD3 negative regulation; (**d**) Activating YAP/TAZ pathway, which regulates both SMAD2/3 shuttling and SMAD-dependent transcriptional activity; (**e**) Controlling myocardin-related transcription factor A (MRTF-A) localization to mediate the SMAD-dependent transcriptional activity.

Whether TGFβ signaling affects actin cytoskeleton is well established, the reciprocal contribution of actin dynamics on TGFβ signaling regulation is still not enough explored. In this section, we will discuss the specific interactions of actin-related functions with TGFβ pathway, from ECM to the nucleus (Figure 1) passing through the receptor trafficking.

### 5.1. TGFβ Ligand Activation: Mechanical Cues

Differently from most of the growth factors that are ready to function upon secretion, TGFβ is typically secreted and stored in the ECM as a latent non-active form. This includes a signal peptide (N-terminal latency-associated peptide LAP), and a C-terminal mature TGFβ, which corresponds to the mature active cytokine monomer [110,111]. The presence of LAP prevents binding of TGFβ to its receptors, impeding signaling function. After secretion, both LAP and mature TGFβ1 homodimers are non-covalently associated in the small latent TGFβ complex (SLC). In most cases, LAP is further covalently associated in the ECM with a latent TGFβ-binding protein (LTBP), to create the large latent complex (LLC), an ECM reservoir of TGFβ. The activation of TGFβ requires the release of the LLC from the ECM and further proteolysis/deformation of LAP to release active TGFβ [13,112].

The best-understood mechanism of active TGFβ release from LLC involves the interaction with a specific subset of integrins, and several RGD-binding integrins are able to activate latent TGFβ through binding to this site [113,114]. Contractile force generated by actin cytoskeleton is required for αvβ6 integrin-mediated activation of TGFβ1 [115]. Tensile force is transmitted by cytoskeleton to integrins, which shift towards an active configuration that favors TGFβ1 release [116]. The inhibitor of actin polymerization cytochalasin D abrogates αvβ6-mediated TGFβ1 activation [115], whereas G-protein coupled receptor agonists induce αvβ6-mediated TGFβ activation. Downstream to G-protein coupled receptor agonists, RhoA and Rho kinase are activated, leading to cytoskeletal reorganization that generates cellular tension, which is transmitted to the cytoplasmic domains of the αvβ6 integrin, and, in turn, activates the large latent TGFβ complex [117,118,119].

TGFβ activation engages a dynamic reciprocity with ECM, and a stiffer ECM increases the free available TGFβ and its activation (Figure 1a), in turn driving a positive feedback by inducing matrix synthesis and stiffness, with pathological consequences [120]. Mechanistically, increased stiffness induces integrin clustering, and, in turn, the activation of Rho family GTPases, which stimulate actin remodeling. This integrin-actin linkage enables cell to sense ECM stiffness and regulates TGFβ signaling (Figure 1a) [40].

### 5.2. ECM-Driven SMAD Intracellular Localization and Transcriptional Activity

Noteworthy, the dynamic remodeling of the actin cytoskeleton is also involved in the localization and activation of SMADs (Figure 1b–e).

In the basal state, SMAD proteins constantly shuttle between the cytoplasm and the nucleus. Once phosphorylated by the receptors, nuclear SMADs activate or repress the transcription of several genes. The constant shuttle of SMAD proteins in to and out of the nucleus also relies on their interaction with the cytoskeleton. Below, some mechanisms of cytoskeleton-related SMAD shuttle are described.

The lamin-binding protein LEMD3 (MAN-1) inhibits TGFβ signaling by binding SMAD2/3 and promoting their dephosphorylation and nuclear export [121,122]. An interesting study by Chambers et al. has shown that the interaction of LEMD3 with SMAD2/3 is negatively regulated by ECM stiffness and antagonized by actin polymerization, highlighting that a stiffened ECM regulates cell response to TGFβ [123].

Nesprin-2 Giant isoform of the Nesprin family proteins is a component of the LINC complex at NE and participates to the mechanical force generation dependent on F-actin network rearrangements, by binding F-actin through the terminal actin-binding domain (ABD) [124]. Studies of wound healing in nesprin-2 Giant deficient mice have reported delayed wound closure and defects in keratinocyte and fibroblast migration and proliferation [125]. Notably, nesprin-2G deficient fibroblasts show defects in cytoskeleton, with F-actin filaments less regular around the nucleus, and a delayed nuclear SMAD accumulation upon TGFβ stimulation [125].

Actin-mediated mechanotransduction intercepts TGFβ signaling also via YAP/TAZ, effectors of the Hippo pathway, critically linked to actin cytoskeleton dynamics as discussed above. TAZ binds and retains SMAD complexes into the nucleus, coupling SMADs to transcriptional machinery. Cytoplasmic retention of phosphorylated TAZ prevents SMAD2/3-SMAD4 complex from accumulating in the nucleus, and TAZ knockdown leads to TGFβ signaling inhibition [126] (Figure 1d). An interesting study shows a multi-step mechanism integrating epithelial polarity with TGFβ signaling. In polarizing epithelial cells, Hippo pathway activation is an early event that promotes cytoplasmic sequestration of TAZ/YAP and suppression of TGFβ-SMAD activity, while prolonged stimulus leads to the basal-lateral restriction of TGFβ receptors, thus reducing SMAD activation. TGFβ receptor sequestration and YAP/TAZ cytoplasmic retention are distinct events that regulate TGFβ signaling in polarized epithelia [127].

Consistent with the involvement of actin cytoskeleton in cellular response to mechanical cues, several studies support a link between matrix rigidity, Rho-Actin-MRTF signatures and TGFβ pathway (Figure 1e). Although the details of this crosstalk are elusive, MRTFs, and in particular MRTF-A, are key regulators of cytoskeletal genes involved in matrix rigidity and TGFβ1-induced EMT. Indeed, TGFβ induces the translocation of MRTFs in to the nucleus and MRTF/SMAD3 complex activates *slug* transcription [128]. Noteworthy, MRTF-A is implicated in TGFβ1-mediated myofibroblastic differentiation in various cell systems and CAF functions [129,130].

In addition, matrix stiffness regulates cytoskeletal architecture [131], and induces the nuclear localization of MRTF-A [132,133], required for the activation of colon and pulmonary fibroblasts [134,135]. The sum of these findings indicates that ECM stiffness and Actin-MRTF-A signaling are crucial in TGFβ1-induced EMT.

### 5.3. Intracellular Trafficking of TGFβ Receptors

Different mechanisms regulating TGFβ receptor availability on the cell surface have been reported [136]. Membrane localization and trafficking of receptors are dynamically regulated by two distinct endocytic pathways: clathrin-dependent internalization into the early endosome, important for propagating signal through the SMAD-dependent pathway, and internalization in caveolin-1-positive lipid rafts that sequester TGFβ receptors for their degradation [137].

**Figure 2:**
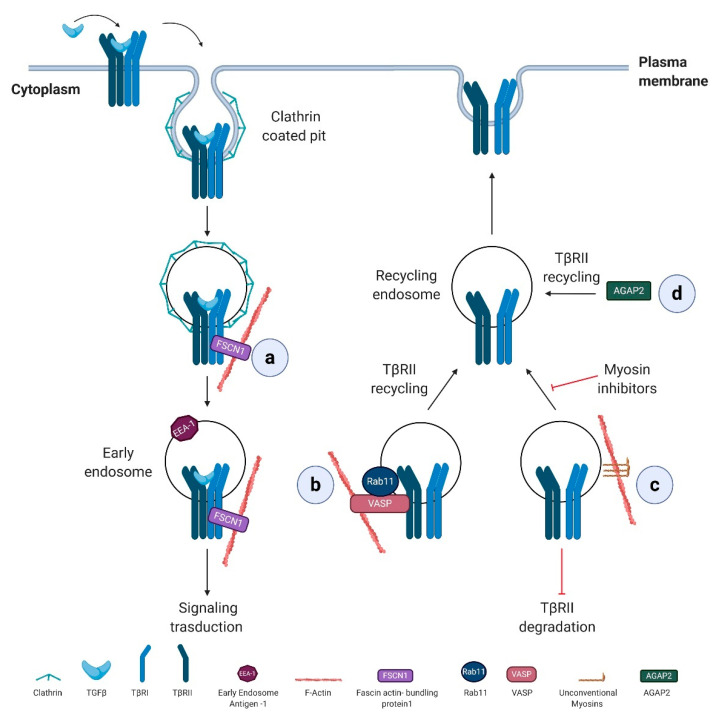
Actin regulatory proteins controlling TGFβ type I receptors (TβRI) and II trafficking. (**a**) The internalized TβRI into clathrin-coated vesicles is linked to the actin cytoskeleton via fascin actin-bundling protein 1 (FSCN1) which promotes the trafficking of internalized receptors from clathrin-coated vesicles to early endosomes and thus TGFβ signaling. (**b**) VASP promotes TGFβ activity by sustaining the complex between Rab11 and TβRII that favors TβRII recycling to the plasma membrane. (**c**) Myosins sustain TGFβ signaling by sustaining the cell-surface expression of TβRII receptor and inhibiting its degradation. (**d**) Arf GAP with GTP-binding protein-like domain, Ankyrin repeat, and PH domain 2 isoform 2 (AGAP2) sustains TGFβ signaling, by directly or indirectly interacting with TβRII and sustaining the recycling of the receptor to the plasma membrane.

Actin dynamics, mainly by regulating intracellular vesicle transport, take part in the complex regulation of TGFβ receptor trafficking via numerous actin regulatory proteins (Figure 2).

Fascin actin-bundling protein 1 (FSCN1), a direct TGFβ/Nodal target gene, is specifically required for the trafficking of TβRI from clathrin-coated vesicles to early endosomes, promoting TGFβ signaling. In particular, FSCN1 specifically interacts with TβRI, and its depletion disrupts the association between receptors and actin filaments and sequesters the internalized receptors into clathrin-coated vesicles (Figure 2a) [138]. Notably, FSCN1 is upregulated by TGFβ/Nodal signaling also in a range of tumor cells and overexpressed in different carcinomas where correlates with the clinical aggressiveness [139], suggesting that FSCN1 sustains TGFβ signaling also during tumor progression.

The monomeric small GTPase Rab11 contributes to TGFβ receptor recycling occurring in early endosome compartments, a process that requires the actin-binding protein VASP. In an experimental liver metastasis mouse model, VASP, by regulating Rab11-dependent TβRII recycling to the plasma membrane, sustains TGFβ signaling activation. Reciprocally, TGFβ stimulation results in VASP upregulation in hepatic stellate cells (HSCs). Overall, TGFβ-mediated activation of HSCs within the hepatic tumor microenvironment is a process essential for metastatic tumor growth in the liver, and VASP takes part to this process by sensitizing hepatic stellate cells to TGFβ effects [140] (Figure 2b).

Unconventional myosins, known to participate in the endocytic trafficking and tether membranes or transport them along the actin cytoskeleton, [141] have been linked to the regulation of TGFβ receptor trafficking. Their specific inhibition reduces cell surface expression of TβRII and promotes receptor degradation (Figure 2c) [142].

TβRII recycling is also affected by Arf GAP with GTP-binding protein-like domain, Ankyrin repeat, and PH domain 2 isoform 2 (AGAP2), a member of Arf GAP (ADP-ribosylation factor GTPase activating proteins) family, critical regulators of membrane trafficking and remodeling of actin cytoskeleton. In the pathophysiology of liver cancer, the depletion of AGAP2 inhibits the recycling of TβRII back to the plasmatic membrane with an important implication in TGFβ-driven pro-fibrotic effects [143]. Proliferation and migration of HSC induced by TGFβ are reduced by AGAP2 depletion and loss of AGAP2 also interferes with TGFβ-dependent collagen type I production (Figure 2d).

### 5.4. SMAD-Dependent Transcriptional Activation

**Figure 3:**
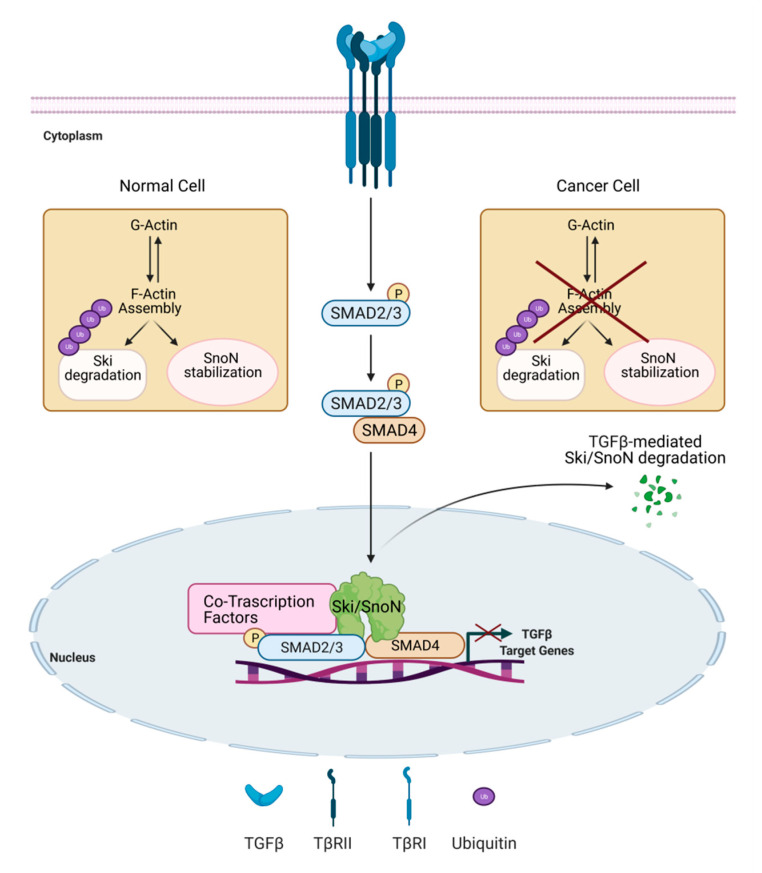
Actin cytoskeleton dynamics control the expression levels of Ski and SnoN, negative regulators of TGFβ signaling. TGFβ signaling controls expression levels of the negative regulators Ski and SnoN, by inducing both their upregulation and their ubiquitin (Ub)-mediated degradation. In normal hepatocytes, actin cytoskeleton dynamics induce Ski protein degradation and SnoN protein stabilization, and might impact TGFβ signaling outcome. This mechanism is lost in hepatoma cells [151,152].

The dynamic of actin cytoskeleton also influences the expression of the negative regulators of TGFβ signaling, Ski (Sloan-Kettering Institute proto-oncogene) and SnoN (Ski-related novel gene) proteins [144]. Ski and SnoN are able to interact with SMAD2/3 and SMAD4 in the cytoplasm and to recruit elements of the repressor machinery on TGFβ target gene promoters [145,146,147]. Notably, the TGFβ/Smad pathway and co-regulators Ski and SnoN clearly control each other by activating positive and negative feedback mechanisms. Ski and SnoN are upregulated by TGFβ signaling and, in turn, they act in a negative feedback manner. Moreover, TGFβ regulates Ski and SnoN levels by inducing their degradation *via* the ubiquitin-proteasome system [148,149]. The alteration in these regulatory mechanisms controls the magnitude and duration of the TGFβ signal and may lead to disease development [150]. Remarkably, changes in actin cytoskeleton dynamics control Ski protein stability and subcellular localization induced by TGFβ in normal hepatocytes mechanisms suggested to be lost in hepatoma cells (Figure 3) [151]. Interestingly, while the inhibition of actin-cytoskeleton rearrangements by cytochalasin D treatment induces a rapid and strong degradation of Ski protein, it stabilizes SnoN protein. This differential actin-cytoskeleton-mediated regulation of the two proteins controls different sets of TGFβ target genes, as demonstrated in normal hepatocytes. On the contrary, in hepatoma cells the actin-mediated modulation of Ski and SnoN protein stability is lost or deeply modified (Figure 3) [152].

### 5.5. Actin-Dynamic Regulators that Control TGFβ Signaling

Several actin regulatory proteins affect TGFβ signaling at different levels. The roles of Rho GTPase family have been extensively described. Herein, we focus on the role of the actin cytoskeleton regulatory protein hMENA, which regulates TGFβ signaling. Of note, *ENAH* gene splicing is regulated by TGFβ. Furthermore, a number of other actin regulatory proteins are reported and summarized in Table 1.

The Rho family of small GTPases, by controlling the polymerization and depolymerization of actin filaments, regulates specific actin cytoskeletal structures, including stress fibers, lamellipodia, and filopodia [49,169]. Rho proteins exert both a positive and negative regulatory role of SMAD-dependent TGFβ signaling. The complete inhibition of Rho activity by C3 exotoxin attenuated SMAD-mediated transactivation and the ectopic expression of a dominant-negative RhoA mutant in neural crest stem cells blocked SMAD2 and SMAD3 phosphorylation and their translocation to the nucleus [153]. On the other hand, the overexpression of RhoB in epithelial cells has been shown to inhibit TGFβ transcriptional program [154]. RhoB but not RhoA overexpression in HaCaT keratinocytes and pancreatic carcinoma cells decreases the expression levels of TβRII and antagonizes the TGFβ-mediated anti-proliferative responses.

Interestingly, antagonistic functions in the regulation of TGFβ signaling have also been reported for the two alternatively spliced isoforms of Rho, ras-related C3 botulinum toxin substrate 1 (RAC1), and RAC1b proteins. Most of the studies have shown a positive interaction between RAC1 and TGFβ signaling [170,171,172], since RAC1 can be activated by TGFβ and may promote the activation of SMAD2. Surprisingly, despite its high structural similarity with RAC1, RAC1b has been shown to be a negative regulator of SMAD signaling, acting as an endogenous inhibitor of RAC1. RAC1b maintains a differentiated epithelial phenotype and prevents the RAC1-driven EMT process [173,174] and cell migration [175]. RAC1b also antagonizes TGFβ dependent EMT by inhibiting p38 and MEK/ERK signaling. Recently, Ungefroren et al. have suggested that RAC1b confers anti-oncogenic properties to pancreatic carcinoma cells not only by acting as an antagonist of RAC1, but also by directly affecting the regulation of main components of TGFβ signal pathway [176]. Ungefroren et al. have also recently revealed an unexpected role of RAC1B in the regulation of TGFβ secretion implicated in cell motility suppression [158]. It is interesting to note that RAC1b increases malignant transformation in response to other EMT-inducers, such as MMP3 [177,178]. Noteworthy, the knockdown of the master regulator of EMT, ESRP1, but not ESRP2, increases the level of RAC1b in head and neck squamous cancer cells [179]. Considering that RAC1 and RAC1b control TGFβ responses in cancer cells in an antagonistic manner, the final outcome of TGFβ signal transduction, in a given tissue or cell type, appears to be strictly dependent on RAC1b/RAC1 ratio [176,180].

Downstream of the Rho GTPases, WAVE3, belonging to the WASP/WAVE family of proteins, activates the Arp2/3 complex, leading to actin polymerization and assembly of actin filaments [181], playing a critical role in cell motility and invasion [182]. WAVE3 expression is highly upregulated by TGFβ in metastatic triple-negative breast cancer cells (TNBCs), where is required for TGFβ-mediated EMT [160]. Noteworthy, the Authors suggest that the TGFβ-induced increase of WAVE3 expression correlates with the functional conversion of TGFβ from a suppressor to a promoter of TNBC development, proposing that therapeutic targeting of WAVE3 may restore the cytostatic activities of TGFβ in late-stage TNBCs [160]. hMENA and its isoforms critically support malignant transformation and progression in different tumors [37,38,183,184,185,186]. Our studies indicated that the alternative splicing of *ENAH* gene generates the alternatively expressed epithelial hMENA^11a^ and mesenchymal hMENAΔv6 isoforms, which contribute to isoform-specific actin cytoskeleton organization, crucial in activating signaling pathways related to immunosuppressive TME such as TGFβ, β1 integrin, and AXL-GAS6 signaling [38,186,187]. hMENA spliced variants have been linked to TGFβ-induced EMT process and TGFβ is involved in the regulation of splicing of *ENAH* gene. TGFβ inhibits the expression of the epithelial splicing factor epithelial splicing regulatory proteins 1/2 (ESRP1/2) and primes the switching of hMENA isoforms by excluding the exon 11a in mammary epithelial and breast cancer cells [33,34,188,189,190]. In addition, TGFβ induces the expression of polypyrimidine tract-binding protein 1 (PTBP1), involved in exon 11a skipping, enhancing migration and invasion of lung cancer cells as well as EMT features in A549 cells [191]. Interestingly, loss of CDC-like kinase 2 (CLK2), kinase linked to the splicing factor RBFOX2, has been shown to activate EMT and TGFβ signaling pathway [192]. Notably, our group has demonstrated that TGFβ1 treatment downregulates hMENA^11a^ expression in pancreatic cancer cell lines, which we have proposed as a prerequisite for EMT [38]. Differently, TGFβ1 upregulates hMENA and the mesenchymal pro-invasive hMENAΔv6 isoform, implicated in SMAD2 phosphorylation and TGFβ1-induced EMT [38]. Noteworthy, hMENA/hMENAΔv6 overexpression defines a CAF subtype with pro-tumoral functions and hMENA expression in CAFs is able to regulate both autocrine and paracrine TGFβ signaling, leading to the regulation of TGFβ-mediated crosstalk between CAFs and tumor cells (unpublished data). We also demonstrated that hMENA isoforms interact with ECM/β1 integrin axis by modulating the expression and activation of β1 integrin and the composition of ECM. Indeed, depletion of all hMENA isoforms in lung cancer cells results in abrogation of stress fibers, actin reorganization, and a dramatic cell shape change, affecting the balance between actin polymerization and depolymerization, that results in F-actin decrease. As a consequence, hMENA affects MRTF-A subcellular localization, SRF activity, and in turn the expression of its target gene β1 integrin.

From a clinical point of view, the pattern of hMENA isoform expression associates with clinical outcome in pancreatic and lung cancer patients [37,38,193].

We have demonstrated that in tumor tissues of early NSCLC patients, the presence of the epithelial-associated isoform, hMENA^11a^ is associated with a low expression of fibronectin in the stroma, and that these tumor features identify patients with better prognosis [186].

The actin regulatory protein Zyxin directly interacts with-actinin and Ena/VASP proteins to dock them to actin filaments [194,195]. Zyxin primarily localizes to focal adhesion, where serves as a docking protein involved in the regulation of cell-extracellular matrix adhesion and in the mechano-transduction process [196]. Several studies pointed at a significant role of zyxin in both physiological and pathological TGFβ1-mediated EMT. In a context of pathological EMT, zyxin has been shown to be not only a functional target of TGFβ, but also an effector of the TGFβ signaling regulation. Indeed, in lung cancer cells, it controls cell motility through the modulation of cell adhesion and expression of integrin α5β1 [161]. Zyxin-knockdown in MDA-MB-231 breast cancer cells abrogates the TGFβ-mediated E-cadherin downregulation and impairs TGFβ-induced cell motility, supporting the notion that zyxin controls TGFβ-induced migration [162]. Recently, high zyxin expression has been associated with a poor prognosis in glioblastoma multiforme patients [197].

A tight association between actin cytoskeleton dynamics and TGFβ1-induced EMT program has also been evidenced for cofilin-1 activity, crucial for the regulation of cell migration and invasion [198]. Cofilin is directly involved in the actin polymerization and remodeling dynamics in response to extracellular signals, including TGFβ and has been firstly identified as a SMAD-independent intracellular effector of TGFβ signaling in prostate cancer cells [199]. Collazo et al. found that Cofilin1 activity coordinates the responses to TGFβ needed for cancer cell migration and metastasis in murine and human prostate cancer [163]. In gastric cancer cells, Wang et al., proved the role of Cofilin in actin-mediated TGFβ-induced EMT [164]. In addition, in colorectal cancer (CRC) Cofilin-1 has been shown to augment the cell–cell disassembly, migration, invasion, and focal adhesion organization during TGFβ-induced EMT. Interestingly, in urothelial cancer, Hensley et al., found a close association of high nuclear localization of Cofilin-1 with increased tumor stage and progression, and have suggested that Cofilin-1 involvement in EMT may be due to its ability to control gene expression by regulating actin organization in the nucleus [200].

Filamin has been identified as a SMAD-binding protein, and Filamin-deficient melanoma cells showed an impaired SMAD2 phosphorylation [166]. Its role as a scaffold to connect the actin cytoskeleton with a multitude of proteins involved in signal transduction has been extensively reported [201,202,203,204]. Due to its ability to interact with SMAD proteins, Filamin has been proposed to function as an anchor protein able to control SMAD protein localization near the cell surface receptors or to keep SMAD conformation, allowing TβRI-mediated phosphorylation [166]. Although Filamin has been originally identified as a tumor promoting factor, prognostic value is mainly dependent on its subcellular localization and binding with different proteins [205]. High Filamin levels have been shown to be predictors of poor patient outcome in several tumors including melanoma, breast, and glioblastoma multiforme [206]. Whereas, the Filamin A isoform has been shown to inhibit tumor progression by regulating breast cancer type 1 susceptibility protein (BRCA1) expression in human breast cancer [207].

An interesting role for actin-binding proteins in the epigenetic regulation of TGFβ signaling has been shown for Profilin 2 (Pfn2). It prevents nuclear translocation of HDAC1 and suppresses its recruitment to SMAD2 and SMAD3 promoters, thus leading to transcriptional activation of SMAD2 and SMAD3. Moreover, Pfn2 correlates with SMAD3 expression in human lung cancers, where its overexpression promotes lung cancer growth and metastasis [167]. On the other hand, the loss of profilin 2 contributes to enhanced EMT and metastasis of colorectal cancer. Thus, the prognostic value of Pfn2 is still controversial [208,209].

Among actin regulatory proteins which nucleate the assembly of unbranched actin filaments, the Formins and in particular the two Formin family members, diaphanous-related formin 1 and 3 (DIAPH1 and DIAPH3) have a crucial role in TGFβ-induced EMT in several tumor cells (i.e., lung, mammary, and renal epithelial cells) [168,210]. The inhibition of Formins, and not of Arp2/3 complex, completely blocked both TGFβ-mediated cell transcription and morphological changes as suggested by their ability to increase the activity of SRF [211,212].

## 6. Conclusions and Perspectives

Herein, we highlight how actin dynamics and TGFβ signaling act in tissue homeostasis and how their deregulation leads to fibrosis and cancer. Key proteins involved in the actin cytoskeleton described in this review are linked to TGFβ signaling and TGFβ-mediated EMT. Our effort was to envision how the deregulation of pathways from ECM to the nucleus involves actin dynamics, contributing to TGFβ-mediated cancer progression. While substantial insights in the field were obtained, new layers of complexity and regulation continue to be discovered.

For progress to occur in the understanding of the crucial role of actin dynamics in cancer, a significant effort needs to be made to identify master regulators of TGFβ pro-tumor activity mediated by actin cytoskeleton dynamics. The comprehension of these mechanisms during cancer progression and overall in therapy resistance will be a future cornerstone for novel TGFβ-directed therapies in the new era of immuno-oncology.

## Figures and Tables

**Table 1:** Actin regulatory proteins affecting TGFβ signaling.

Actin Regulatory Protein	Role in TGFβ Signaling	Function	References
**RhoA**	SMAD2/3 phosphorylation/activation	Modulates SMAD Signaling during TGFβ-induced Smooth Muscle Differentiation	[153]
**RhoB**	SMAD-mediated transcriptional activity	Antagonizes TGFβ transcriptional program	[154]
	Transcription of TβRII	Antagonizes TGFβ-mediated anti-proliferative and transcriptional responses in keratinocytes and pancreatic carcinoma cells	[155]
**Rac1**	SMAD2 phosphorylation/activation	Antagonizes TGFβ1-mediated growth inhibition and sustains TGFβ1-induced cell migration in pancreatic ductal adenocarcinoma cells	[156]
**Rac1b**	SMAD2/3 phosphorylation/activation	Inhibits TGFβ1-induced cell motility in pancreatic ductal epithelial cells	[157]
	Synthesis and secretion of TGFβ1	Suppresses cell motility	[158]
	TGFβ1-mediated p38MAPK and MEK-ERK activation	Antagonizes TGFβ1-dependent EMT	[159]
**WAVE3**	Transcription of EMT-related genes	Regulates TGFβ-mediated EMT and growth and metastasis of triple-negative breast cancer cells	[160]
**hMENA/hMENAΔv6**	SMAD2/3 phosphorylation/activation	Regulates TGFβ-mediated EMT in PDAC cells	[38]
**Zyxin**	Regulation of TGFβ-induced integrin α5β1 expression	Regulates cancer cell motility and EMT during lung cancer development and progression	[161]
	Regulation of EMT-related protein	Mediates cooperation between Hippo and TGFβ signaling pathways	[162]
**Cofilin**		Drives Cell-Invasive and Metastatic Responses to TGF-β in Prostate Cancer	[163]
	Regulation of EMT-related morphology and protein expression	Sustains TGFβ-mediated EMT in gastric cancer cells	[164]
		Sustains migration and invasion during EMT of CRC cells	[165]
**Filamin**	SMAD localization	Sustains SMAD-dependent TGFβ signaling	[166]
**Profilin 2**	Epigenetic regulation of SMAD2 and SMAD3	Promotes lung cancer growth and metastasis	[167]
**Formins**	SMAD-mediated transcriptional activity	Sustains TGFβ-mediated EMT	[168]
